# Pasta from Red Lentils (*Lens culinaris*): The Effect of Pasta-Making Process on Starch and Protein Features, and Cooking Behavior

**DOI:** 10.3390/foods11244040

**Published:** 2022-12-14

**Authors:** Andrea Bresciani, Daniela Erba, Maria Cristina Casiraghi, Stefania Iametti, Alessandra Marti, Alberto Barbiroli

**Affiliations:** Department of Food, Environmental and Nutritional Sciences (DeFENS), Università degli Studi di Milano, Via G. Celoria 2, 20133 Milan, Italy

**Keywords:** pulses, proteins, pasta, extrusion, extrusion-cooking, pre-gelatinization, in vitro starch digestibility

## Abstract

The effect of pasta-making processes on starch and protein features, as well as cooking behavior, and nutritional properties (i.e., resistant starch and starch in vitro digestibility) were assessed. Pasta from raw red lentils (R) was prepared by conventional extrusion (C_R) and extrusion-cooking (EC_R), whereas heat-treated red lentils (HT) were processed into pasta by conventional extrusion (C_HT). A “high protein” and “high fiber” pasta was prepared. Using HT was effective in increasing the luminosity (that was about 88, 91, and 96 for EC_R, C_R, and C_HT, respectively), and decreasing the presence of defects on the pasta surface (heterogeneity was 5%, 36%, and 45% for C_HT, EC_R, and C_R, respectively). Heat treatment on grains or flour significantly increased starch susceptibility to α-amylase (6.6, 7.4, and 8.6% for C_R, C_HT, and EC_R, respectively) and decreased the final viscosity (from 335 BU in C_R to 287 and 291 BU in EC_R and C_HT), resulting in a significant increase in starch digestibility (slowly digestible starch was about 41, 27, and 26% in C_R, C_HT, and EC_R, respectively). As regards proteins, the main effect on their structure was observed in C_HT, where the cooking behavior was much improved and cooking losses were lowest (5.7%). On the other hand, protein and starch organization in EC_R might have accounted for pasta resistance in overcooking.

## 1. Introduction

Climate changes and their impact on water and land resources are increasing consumer awareness of the impact of their diet on the planet. From this awareness comes the request for reformulating staple foods, for example, by totally or partially replacing conventional grains with more sustainable crops. Among the latter, pulses play a key role in promoting a sustainable healthy diet, thanks to their positive impact on soil, environment, and individual’s health and well-being. However, food reformulation is not so easy, especially if the structural properties of the “conventional” product are taken as references for consumer acceptability. In the case of pasta—the simplest cereal-based product in terms of formulation and processing—the structural properties are provided by the protein and starch organization. Understanding the effects of formulation and/or processing conditions on molecular features seems to be strategic in defining the quality of the final product. Throughout the decades, structure-quality relationships have been studied in refined [1] and wholegrain [2,3] wheat pasta, where gluten plays a key role in assuring the typical “al dente” texture of the cooked product, as well as in gluten-free pasta [4,5,6], where starch modifications are strategic in creating a cohesive structure. On the contrary, very little information is available so far on the role of starch and protein in defining the quality of pulse pasta—the latest innovation in the pasta sector—containing about 60% of starch and 20% of proteins [7].

By addressing starch and protein features in commercial pulse pasta, Bresciani et al. [7] pointed out that a stiff protein network resulted in better cooking behavior. Despite providing insights on how protein molecular organization may have affected cooking behavior, the study did not address processing conditions, that were unknown due to property issues in the case of commercial samples.

With the objective of elucidating the effect of processing on starch properties and cooking behavior, Bresciani et al. [8] prepared pasta from yellow lentils by using two approaches: conventional extrusion (as for semolina pasta) and extrusion-cooking process (as for gluten-free pasta), in which flour is subjected to thermal and mechanical treatment to modify starch functionality [9]. The results highlighted that pasta from extrusion-cooking exhibited higher stability during cooking and resistance to overcooking, resulting in firm pasta. Since starch is the main component even in pulse pasta, the cooking behavior of yellow lentil pasta was related to the structural modification induced by the shear and thermal stresses on starch granules [8], neglecting the potential role of proteins.

As for gluten-free cereals, gelatinization of pulses can occur either during the pasta-making process (e.g., in the extrusion-cooking process) or even before (i.e., by using pre-gelatinized flours) [9]. In a recent study, it was found that pre-gelatinization modified both starch and protein organization in red lentils [10]. However, up to about 40% of gelatinized starch does not impact the cooking quality of pasta produced on a lab scale, but might negatively impact the nutritional properties of cooked pasta by decreasing its resistant starch content and increasing the starch hydrolysis index [11].

Although starch is the main component in pulses, the effects of pasta-making processing on overall protein organization and its role in defining pasta cooking behavior should not be neglected. In this context, the aim of the present study was to provide insights on the effect of processing conditions (i.e., conventional extrusion of raw and heat-treated red lentil flour and extrusion-cooking of raw flour) on both starch and protein features also in relation to pasta quality.

## 2. Materials and Methods

### 2.1. Raw Materials

Flours from raw (R) and heat-treated (HT) red lentils (*Lens culinaris*; starch: 51%; protein: 22%) were provided by an Italian mill. Raw material characterization was reported in previous work [10].

### 2.2. Pasta-Making Process

Flour from raw red lentils (100%) was mixed with water (30% final moisture content) and processed into pasta by means of two approaches, conventional extrusion (C_R) and extrusion-cooking (EC_R). Flour from heat-treated red lentils was processed only by conventional extrusion (C_HT) [8]. When required, pasta was ground (particle size < 250 µm) using a laboratory mill (IKA Universalmühle M20; IKA Laborteknic, Staufen, Germany). The chemical composition of pasta is reported in Supplementary Table S1, with all the samples showing high protein (more than 20% of the energy value of the product) and fiber (more than 6 g per 100 g) content, therefore allowing “high in protein” and “high in fiber” claims (EC No. 1924/2006) [12]. Moisture, ash, proteins, and lipids were determined according to the approved methods AACC 44-15, 08-12, 46-12, and 30-10, respectively [13]. The amount of total, soluble and insoluble dietary fiber was determined according to AACC 32-07.01 method [13]. Carbohydrates were calculated by difference.

### 2.3. Surface Characteristics of Uncooked Pasta

The color of ten pieces of uncooked pasta was measured using a reflectance color meter (CR 210, Minolta Co., Osaka, Japan). The sample was covered with a black box and the lightness and saturation of the color intensity were measured and expressed in the CIE L* a* b* color space.

Images of uncooked pasta were acquired at 300 dots per inch using a scanner (Epson Perfection 550 Photo, Seiko Epson Corp., Suwa, Japan). Surface heterogeneity was assessed on ten pieces of uncooked pasta. The images were processed using the Image Pro-Plus 4.5.1.29 software (Media Cybernetics Inc., Marlow, UK). Surface heterogeneity was calculated on the images converted in 8-bit greyscale. Heterogeneity is defined as the fraction of pixels whose intensity value deviates more than 10% compared to the average intensity of the entire image. A heterogeneity value equal to zero corresponds to a homogeneous surface (smooth surface); on the other hand, a value equal to one corresponds to a heterogeneous surface (rough surface).

### 2.4. Starch Properties

#### 2.4.1. Susceptibility to α-Amylase Hydrolysis

Starch susceptibility to α-amylase was measured on uncooked pasta according to the AACC 76-31.01 method [13]. In this method, quickly accessible starch was hydrolyzed (10 min at 40 °C) to maltosaccharides and limited dextrins by fungal alpha-amylase (product code: E-ANAAM; Megazyme, Wicklow, Ireland). Subsequently, an excess of amyloglucosidase (product code: E-AMGDF; Megazyme, Wicklow, Ireland) was used to convert released oligosaccharides to glucose, which was then quantified spectrophotometrically. Data are referred to on 100 g of total starch, which was determined using the AACC 76-13.01 method [13].

#### 2.4.2. Pasting Properties

The pasting properties of uncooked pasta were evaluated in a Micro Visco-Amylo-Graph (MVAG; Brabender GmbH., Duisburg, Germany) [8]. One representative curve for each sample was reported.

### 2.5. Protein Properties

#### 2.5.1. Susceptibility to Tryptic Hydrolysis

A suspension of 75 mg of ground samples in 6 mL of 50 mM sodium phosphate, 0.1 M NaCl, and pH 7.0 were mixed with 150 µL trypsin solution (2 mg/mL in 20 mM sodium acetate, pH 4.5) and incubated at 37 °C for 40 min. The hydrolysis was stopped by adding 6 mL of 20% (*w*/*v*) trichloroacetic acid (TCA). The suspension was then centrifuged (13,000× *g* for 10 min) to remove the precipitated undigested proteins. Soluble peptides were quantified by reading the absorbance of the supernatant at 280 nm versus a blank consisting of phosphate buffer and TCA [10].

#### 2.5.2. Sodium Dodecyl Sulfate-Polyacrylamide Gel Electrophoresis (SDS-PAGE)

Samples were prepared by heating at 100 °C for 10 min a suspension consisting of 2 mg of ground samples, 100 µL of water, and 100 µL of denaturing buffer. Denaturing buffer was as follows: 0.125 M Tris-HCl, pH 6.8, 50% (*v*/*v*) glycerol, 17 mg/mL SDS, 0.1 mg/mL bromophenol blue; 2.5% (*v*/*v)* 2-mercaptoethanol (2-ME) was also added for reducing condition. The denatured suspensions were centrifuged (5000× *g* for 3 min) to remove insoluble debris. The electrophoretic separations were carried out on the clear supernatants, by using a 12% acrylamide gel run in a Miniprotean II apparatus (Bio-Rad Laboratories, Hercules, CA, USA). The gels were stained with Coomassie Blue.

#### 2.5.3. Differential Solubility

Protein solubility was evaluated in native and denaturing/reducing conditions. For each sample, three aliquots of the ground sample (150 mg) were suspended respectively in (i) 6 mL of saline buffer (50 mM sodium phosphate, 0.1 M NaCl, pH 7.0), (ii) 6 mL of saline buffer containing 4 M urea, and (iii) 6 mL of saline buffer containing 4 M urea and 10 mM dithiothreitol (DTT). The suspensions were incubated for 90 min at 25 °C under mild stirring and subsequently centrifuged (10,000× *g* for 20 min, 25 °C) [14]. Proteins solubilized in the supernatants were quantified by a dye-binding method [15].

#### 2.5.4. Quantification of Accessible Thiols

Quantification of accessible thiols (-SH) was carried out by exploiting the ability of the reagent 5,5-dithio-bis-nitrobenzoate (DTNB) to react with the exposed -SH groups of cysteine [16]. Ground samples (50 mg) were suspended in 6 mL of 50 mM sodium phosphate, 0.1 M NaCl, pH 7.0, containing 0.2 mM DTNB, and incubated for 1 h g at 25 °C under shaking. The suspensions were then centrifuged (13,000× *g* for 15 min, 20 °C), and the number of accessible thiols (µmol -SH/g pasta) were calculated from the absorbance of the clear supernatant at 412 nm (ε = 13,600 M^−1^ cm^−1^) read against a DTNB blank [10].

#### 2.5.5. Front-Face Fluorescence

Intrinsic and extrinsic fluorescence were assessed by front-face (solid-state) measurements [17]. Conformational changes upon solvation and kneading were studied by intrinsic (tryptophan) fluorescence. Samples were prepared by adding to the ground raw pasta sample (1 g) enough water to achieve a final moisture content of 30% (i.e., the same moisture content used for pasta making). The mixture was kneaded for 2 min by using a glass stirring rod, and then loaded in the front-face fluorescence cell to monitor tryptophan fluorescence (emission range 300–420 nm, excitation wavelength 280 nm, scan speed 50 nm/min, emission and excitation slits 2.5 nm). Changes in protein surface hydrophobicity were assessed in extrinsic fluorescence studies, by using the hydrophobic fluorescent probe 1,8-anilinonaphthalene-sulfonate (ANS) [17]. Raw pasta samples were prepared as reported above for tryptophan fluorescence, except that a suitable volume of an ANS stock solution (5 mM in water) was added instead of water to achieve a final probe concentration of 0.5 mM in the resulting dough. ANS fluorescence intensity was monitored at 460 nm (excitation wavelength 390 nm, emission and excitation slits 2.5 nm). All of the measures were performed at 25 °C in a LS50 B Perkin-Elmer Luminescence Spectrometer (PerkinElmer Inc., Waltham, MA, USA). 

### 2.6. Cooking Behavior

Pasta water absorption, cooking loss, and instrumental texture (using a Kramer shear cell with 10 blades) were measured [8] at both optimal cooking time and overcooking. The optimal cooking time was 6.5 min for all the samples, and it was evaluated by a trained panel composed of ten people who tasted the firmness of pasta every 30 s starting from 3 min of cooking. Overcooking (i.e., 8 min) was calculated as a 25% increase with respect to optimal cooking time.

### 2.7. Starch Properties on Cooked Pasta

#### 2.7.1. Resistant and Total Starch Contents

Each pasta sample was cooked at the optimal cooking time. Cooked pasta was immediately minced (0.9 cm particle size) to simulate mastication and analyzed. Total and resistant starch were assessed by AACC 32-40.01 and AACC 76-13.01 methods, respectively [13].

#### 2.7.2. In Vitro Starch Digestibility

Rapidly (RDS) and slowly (SDS) digestible starch fractions were determined using the method of Englyst [18]. Glucosereleased at 20 min and between 20 and 120 min of incubation with pepsin (P7000, from gastric mucosa) and then with a mixture of hydrolytic enzymes (P7545, pancreatin from porcine pancreas; A7095, amyloglucosidase from *Aspergillus niger*, from Sigma Chemical Co., St Louis, MO, USA) was measured by High Performance Liquid Chromatography [19]. Data were expressed as a percentage of SDS and RDS on total available starch.

### 2.8. Statistical Analysis

Results of susceptibility to tryptic and amylase hydrolysis, protein solubility, accessible thiols, front-face fluorescence, as well as pasting properties, total, resistant, slowly, and rapidly digestible starch were expressed as mean ± standard deviation of three replicates. Data on cooking behaviour come from five independent cooking trials.

Analysis of variance (ANOVA) was carried out using Statgraphics Plus 5.1 (StatPoint Inc., Warrenton, VA, USA) to determine significant (*p* < 0.05) differences among the samples. When a factor effect was found to be significant, significant differences among the respective averages were determined using Tukey’s honest significant difference (HSD) test.

## 3. Results and Discussion

### 3.1. Surface Characteristics of Uncooked Pasta

**Figure 1:**
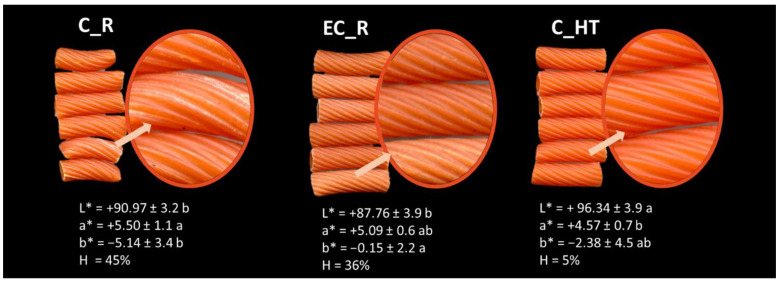
Images and surface characteristics of uncooked pasta from red lentils.

Images of pasta are reported in Figure 1, together with color and heterogeneity analysis. The evaluation of pasta quality by consumers starts at the grocery store, where surface characteristics (i.e., color and absence of defects) and labels play an important role in driving consumer choice [20]. Both the raw materials and the type of process significantly impacted on surface characteristics of red lentil pasta, with C_HT exhibiting the highest luminosity and the lowest heterogeneity value, which is an objective measurement of the white specks visible on the surface as the effect of incomplete flour hydration.

Pre-treatment of raw material was effective in decreasing the white specks and may be ascribed to the improved hydration properties and dough-forming capacity of heat-treated red lentils compared to the raw (untreated) sample, as a result of both starch gelatinization and protein denaturation [10]. This pre-treatment also affected the yellowness, which was twice that of pasta obtained with the same process from untreated materials.

The pasta processing also affected the heterogeneity and yellowness, as shown by the data related to C_R and EC_R. Modification of the pasta surface characteristics upon heat treatment (by the extrusion-cooking process) was also observed in pasta from yellow lentils produced with an extrusion-cooking process starting from native flour [8].

### 3.2. Starch Properties

#### 3.2.1. Starch Susceptibility to Alpha-Amylase

As reported elsewhere [8,21], the “starch susceptibility index” provides insights into the effect of the milling process on starch damage when applied to native flour; whereas, in processed foods, it provides information on the effect of processing on starch organization. However, this index evaluates the accessibility of the sample to a short (i.e., 10 min) exposure to α-amylase, and thus provides information on the molecular organization only of the outermost regions of the granules.

The amount of starch susceptible to a quick α-amylase hydrolysis followed the order EC_R > C_HT > C_R (Table 1). The highest value in EC_R could be explained by the combination of high shear stress and temperature that modified the physical and structural organization of starch granules as observed in both rice [22,23] and yellow lentils [8].

#### 3.2.2. Pasting Properties

**Figure 2:**
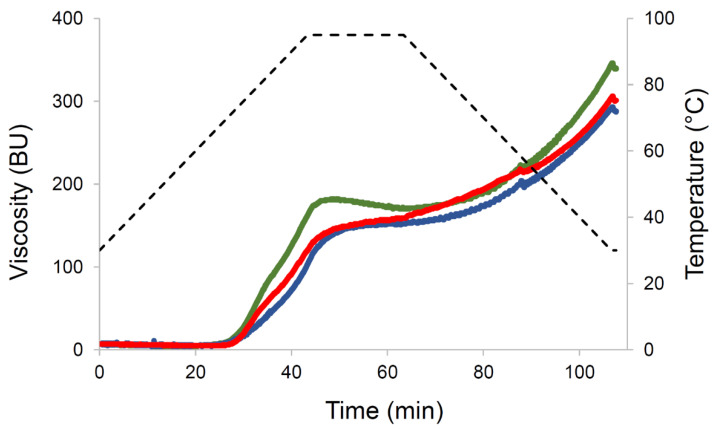
Pasting profile of pasta from red lentils. The green line is pasta from raw red lentils by conventional extrusion (C_R); the blue line is pasta from raw red lentils by extrusion-cooking (EC_R); the red line is pasta from heat treated red lentils by conventional extrusion (C_HT); the dotted black line is temperature profile. BU is Brabender Unit.

The pasting profiles of pasta are reported in Figure 2, and the related indices are listed in Table 1. The pre-treatment of the raw material (either on the grains to produce HT or in the tank during the extrusion-cooking process) and further conventional extrusion delay starch gelatinization during the MVAG test. This is made evident by the increase in pasting temperature (Table 1) as well as by the decrease in the slope of the curve during the heating phase (Figure 2). Moreover, upon cooling, a lower final viscosity was measured in EC_R and C_HT compared to C_R. Two phenomena might account for the differences observed among the samples: partial starch gelatinization and/or mechanical degradation can be expected in EC_R and C_HT samples; but it should also be considered that thermally modified starch granules tend to reorganize in a more compact structure during the “natural” cooling of the product so that the ungelatinized starch in EC_R and C_HT were less prone to further gelatinize during the MVAG test.

By applying both conventional extrusion and extrusion-cooking processes on native yellow lentils, Bresciani et al. [8] pointed out that pasta from extrusion-cooking showed a higher pasting temperature, and a lower maximum viscosity than pasta from conventional extrusion. Differences between red and yellow lentils might result in different behavior during processing accounting for the differences between the two studies.

Although pasta behavior during the MVAG test might be due to the occurrence of gelatinization (and consequently to its reorganization upon cooling during processing), the effect of protein on starch swelling should be also considered [1,24].

### 3.3. Protein Properties

#### 3.3.1. Protein Susceptibility to Tryptic Hydrolysis

Pasta susceptibility to the actions of proteolytic enzymes provided insights into the compactness of the protein structure. Indeed, generally, low accessibility to proteases is associated with a native and compact structure, whereas increased accessibility is associated with the flexible structure typical of partially unfolded proteins [25].

**Figure 3:**
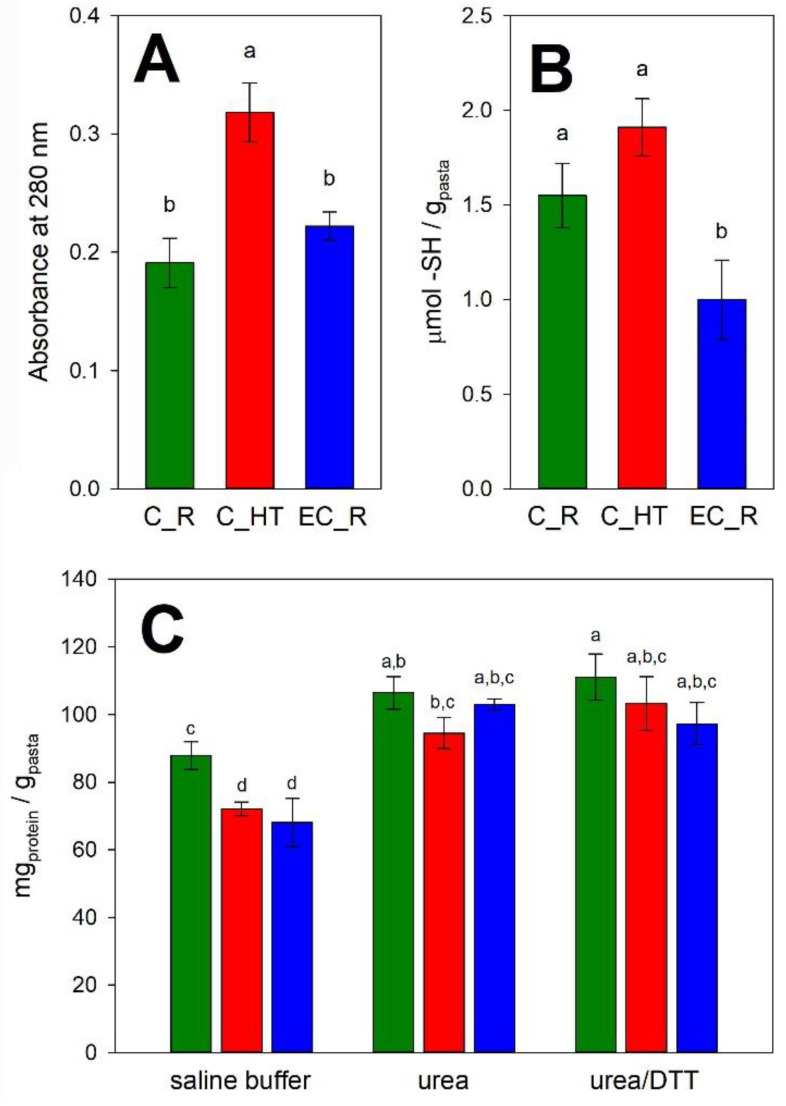
Protein features in pasta from red lentils. Panel (**A**) Protein susceptibility of raw pasta samples to tryptic hydrolysis. Panel (**B**) Amount of accessible protein thiols. Panel (**C**) Protein differential solubility of pasta in non-dissociating (saline buffer), dissociating (urea), and reducing (urea/DTT) buffers. Samples marked with the same letter are not significantly different (Tukey’s HSD; *p* < 0.05). Green: C_R, pasta from raw red lentils by conventional extrusion; red: C_HT, pasta from heat-treated red lentils by conventional extrusion; blue: EC_R, pasta from raw red lentils by extrusion-cooking.

The susceptibility to tryptic hydrolysis followed the order C_HT >> EC_R ≈ C_R (Figure 3A), suggesting that the observed structural protein modifications induced by the heat-treatment of flour [10] had an impact in defining conformational pasta structure during pasta-making processing.

#### 3.3.2. Protein Differential Solubility

Information on the presence and nature of protein aggregates in red lentil pasta was obtained by differential solubility approaches, i.e., by evaluating protein extraction in buffers with different dissociating abilities towards covalent and non-covalent interprotein bonds [1]. Compared to the red lentil flour (for which results are reported in Bresciani et al. [10]), all the heat treatments here proposed (either if performed on the raw material or during the extrusion-cooking process) resulted in a decrease in protein extractability in saline buffer (i.e., under non-dissociating condition), indicating that these treatments led to the formation of more aggregated and thus less accessible protein structures. Similar behavior was also detected in rice pasta [5].

In the case of pulse pasta, the highest amount of proteins solubilized in saline buffer (i.e., when the soluble proteins are not involved in the formation of aggregates) was measured in C_R, i.e., pasta obtained by conventional extrusion of raw flour (Figure 3B), whereas no significant differences were observed when the processing involved a thermal treatment, either by using heat-treated flour in a conventional extrusion process (C_HT) or by treating raw flour in an extrusion-cooker (EC_R). Results suggested that the heat treatments promoted the formation of aggregates between somehow unfolded proteins. A similar result was observed after thermally treating red lentil flour [10]. For all the samples, extracted proteins in phosphate buffer were much lower than those solubilized upon the addition of urea, suggesting that the protein aggregates were stabilized mainly by hydrophobic bonds. Indeed, when both urea and DTT were present in the extraction medium, the number of soluble proteins did not increase further (Figure 3B), suggesting that covalent interactions (i.e., disulfide bounds) play a marginal role in stabilizing the aggregates. The low relevance of disulfide bridges can be linked to the low content of sulfur amino acids typical of legumes [26]. Differently, in gluten-free cereals, such as rice, inter-protein disulphides play a fundamental role in the structure of the protein network when heat-treated rice (i.e., parboiled, whose process also promoted starch gelatinization during steaming) was used instead of native rice in rice pasta production [5].

The protein pattern of various pasta samples was characterized by sodium dodecyl sulfate-polyacrylamide gel electrophoresis (SDS-PAGE) in the presence/absence of reductant (Supplementary Figure S1). No differences were observed in the protein patterns of the three pasta samples, either in non-reducing or reducing conditions, confirming that the difference in solubility data may be ascribed to interaction driven by hydrophobic interactions, which occur as a result of conformal changes induced by the technological treatment applied in the different pasta making processes.

#### 3.3.3. Quantification of Accessible Thiols

Further insight into the compactness of the protein matrix came from the quantification of accessible -SH groups on cysteine side chains. As shown in Figure 3C, the number of accessible cysteine thiols followed the order C_HT ≥ C_R > EC_R, confirming the impact of the heat treatment of red lentils on the compactness of the protein matrix and that the effects of extrusion-cooking on protein structural rearrangements were more dramatic than those of conventional extrusion, as previously observed in pasta from parboiled rice [5]. Results in Figure 3C suggested that the extrusion-cooking process influenced the structure of proteins by increasing their compactness, resulting in lower accessibility of the cysteine thiols that became buried in the protein network.

#### 3.3.4. Front-Face Fluorescence

The tryptophan emission maximum recorded in front-face fluorescence upon kneading ground raw pasta samples with 30% water is reported in Table 2. During unfolding, the hydrophobic regions of the protein are exposed to the outside. As the water level increases, the chemical environment of the exposed tryptophan side chains becomes more polar, resulting in a redshift (i.e., an increase in the wavelength) of its maximum fluorescent emission [17]. The emission wavelength in C_HT was higher than in the other samples. Comparing the two processes applied to the raw (untreated) flour, proteins in the extrusion-cooking sample had an emission maximum slightly higher than protein in the conventional extrusion sample, but much lower than in samples from heat-treated red lentils.

When comparing the tryptophan fluorescence data of flours (337.9 ± 0.4 and 340.0 ± 0.3 nm for R and HT, respectively [10]) with those on the corresponding pasta, a further redshift is evident in pasta, likely stemming from further protein denaturation during processing. The treatment that brings about the most pronounced conformational changes on the protein flours is the heat treatment of the raw material combined with conventional extrusion and may provide an explanation at the molecular level of the detected cooking behavior of the corresponding pasta.

A fluorescent probe (i.e., ANS) was used to provide information on the protein surface hydrophobicity: since the probe becomes fluorescent when it binds to the exposed hydrophobic patches, measuring the fluorescence of the samples in the presence of ANS at fixed concentrations provides information on the impact of processing on protein surface hydrophobicity [17]. At 30% moisture content, fluorescence in excess of ANS - which is proportional to the overall surface hydrophobicity—is highest in C_HT pasta, whereas it does not vary in the other two samples produced from raw flour (Table 2). The comparison with the results on the flours (20.8 ± 0.9 and 13.9 ± 1.0 A.U., for R and HT respectively [10]), showed that for the raw flour the surface hydrophobicity decreased upon the pasta-making process, suggesting the formation of compact protein structures after extrusion and/or drying. In heat-treated flour, the conventional extrusion results in a protein rearrangement that slightly increased the overall surface hydrophobicity. These results underlined the complexity of the systems, where the nature and extent of conformational changes induced by pasta making process (conventional or extrusion-cooking) on proteins depend also on the state of the protein on the raw material (native in the raw flour, unfolded in the heat-treated flour).

### 3.4. Cooking Behavior

Water absorption, cooking loss, and instrumental firmness at the optimal cooking time (6.5 min) and overcooking (8 min) are summarized in Table 3. EC_R showed the lowest water absorption and firmness, but the highest cooking loss. Although the same trend was kept even during overcooking, EC_R was able to maintain its “structure” showing little differences in its behavior when cooking was prolonged by 1.5 min, suggesting greater thermal stability than the other two samples. Protein features (i.e., low thiol accessibility, great protein stabilization by hydrophobic interactions) and starch organization (i.e., high starch susceptibility to α-amylase) in EC_R might account for cooking behavior. Differences between conventional and extrusion-cooking were not detected in the case of yellow lentils [8], pointing out that different raw materials might behave differently during processing. Finally, C_HT pasta showed lower water absorption and cooking loss but a similar texture to the C_R sample (Table 3). When the pasta was produced at lab scale [11], pre-treatment of red lentils did not affect the quality of the product, likely due to the low pressure used during the pasta-making process.

Commercial pasta from red lentils exhibited a water absorption higher than 100%, cooking loss between 7.5 and 8.1 g/100 g, and firmness between 398 and 437 N [7]. Differences among the studies might be due to differences in processing conditions, including pasta shape. A commercial pasta from red lentils of a similar shape to our experimental pasta showed, at an optimal cooking time of 7 min, a firmness of about 500 N. Thus, although C_R and C_HT showed a low cooking loss, their texture should be considered too firm compared to the commercial samples.

### 3.5. Total and Resistant Starch and In Vitro Starch Digestibility

The pasta-making processes considered in this study did not significantly affect the total and resistant starch content of cooked samples (Table 3). Although the amount of resistant starch in red lentil pasta was higher than that found in gluten-free pasta from rice, corn, and their combinations [3,5,21] our experimental pasta did not reach the resistant starch level of at least 14% of the total starch content that would assure the claim of “replacing digestible starch with resistant starch induces a lower blood glucose rise after a meal” [27]. Several approaches can be adopted to increase the resistant starch of products, such as the choice of grains with high amylose content or the application of chemical, physical or enzymatic treatments on flours [28,29].

As regards starch digestibility, RDS has been reported as related to glycemic responses, whereas SDS is inversely correlated to insulin demand [30]. In red lentil pasta, RDS followed the order C_R < EC_R = C_HT (Table 3), indicating that heat treatment significantly increased the starch fraction digested within 20 min of hydrolysis. Consistently, SDS appeared higher in C_R than in the other samples. These results are consistent with starch susceptibility to α-amylase in the uncooked samples, and sustain the food matrix rearrangement due to the heating treatments. A health claim on SDS has been approved by the European Food Safety Authority [31]. Specifically, the claim may only be used on foods in which digestible carbohydrates provide at least 60% of the total energy and in which at least 55% of those carbohydrates consist in digestible starch, which in turn consists of at least 40% of SDS. Among our samples, only C_R could potentially meet such criteria, with 62% of energy from carbohydrates, 97% of starch/total carbohydrates, and 40.7% of SDS/available starch, meeting the claims under EU No 851/2013.

## 4. Conclusions

Heat treatments, either on the raw material (pre-gelatinization) or during the process (extrusion-cooking), are largely applied in the production of gluten-free pasta in order to improve its overall quality. Our data on starch and protein organization, as well as on appearance and cooking behavior, indicate that heat treatment has an important role in defining the overall properties of red lentil pasta. In this frame, the observed pasta properties strictly depend on the nature of the heat treatment. Indeed, pasta produced by conventional extrusion of heat-treated grains (C_HT) showed protein and starch organization that positively affect its cooking behavior (low cooking loss) and appearance (low heterogeneity) in comparison with pasta produced by conventional extrusion (C_R) and by extrusion-cooking (EC_R) of untreated lentils. From a nutritional standpoint, a “high protein” and “high fiber” pasta was obtained. Moreover, although both heat treatments lead to an increase in rapidly digestible starch, the level of resistant starch is not affected by the process. Further studies will focus on the effect of processing on consumers’ acceptability.

## Figures and Tables

**Table 1:** Starch susceptibility to α-amylase and pasting properties of pasta from red lentils.

Samples	Starch Susceptibility to α-Amylase (g/100 g d.b.)	Pasting Temperature (°C)	Maximum Viscosity (BU)	Final Viscosity (BU)	Setback (BU)
C_R	6.6 ± 0.3 c	71 ± 0.7 b	192 ± 13.4 n.s.	335 ± 5.7 ab	162 ± 9.9 n.s.
EC_R	8.6 ± 0.2 a	73 ± 0.1 a	158 ± 7.1 n.s.	287 ± 2.1 b	133 ± 9.9 n.s.
C_HT	7.4 ± 0.2 b	74 ± 0.1 a	158 ± 2.1 n.s.	291 ± 12.7 b	137 ± 7.8 n.s.

Mean (*n* = 3) ± standard deviation followed by different letters in the same column are significantly different (Tukey’s HSD; *p* < 0.05). n.s., not significant. d.b., dry basis. BU, Brabender Unit. C_R, pasta from raw red lentils by conventional extrusion; EC_R, pasta from raw red lentils by extrusion-cooking; C_HT, pasta from heat-treated red lentils by conventional extrusion. Pasting temperature is the temperature at which an initial increase in viscosity occurs; maximum viscosity is the maximum viscosity reached during the analysis; final viscosity is the viscosity at the end of the test; setback is the difference between the final viscosity at 30 °C and the viscosity reached at the end of the holding period.

**Table 2:** Front-face fluorescence protein conformational indices of pasta from red lentils.

Samples	Intrinsic Fluorescence (Fluorescence Maximum Wavelength, nm)	Protein Surface Hydrophobicity (ANS Fluorescence Intensity at 460 nm, A.U.)
C_R	339.4 ± 0.4 c	11.5 ± 0.8 b
C_HT	342.0 ± 0.3 a	15.9 ± 0.4 a
EC_R	340.3 ± 0.3 b	11.1 ± 0.8 b

Mean (*n* = 3) ± standard deviation followed by different letters in the same column are significantly different (Tukey’s HSD; *p* < 0.05). C_R, pasta from raw red lentils by conventional extrusion; C_HT, pasta from heat treated red lentils by conventional extrusion; EC_R, pasta from raw red lentils by extrusion-cooking.

**Table 3:** Cooking behavior and starch digestibility of pasta from red lentils.

	C_R	EC_R	C_HT
Cooking performance			
Optimal cooking time (6.5 min)			
Water absorption (g/100 g)	81 ± 1 a	66 ± 1 c	77 ± 1 b
Cooking loss (g/100 g d.b.)	6.3 ± 0.3 b	9.4 ± 0.6 a	5.7 ± 0.4 c
Firmness (N)	668 ± 30 a	548 ± 36 b	637 ± 26 a
Overcooking (8 min)			
Water absorption (g/100 g)	92 ± 2 a	87 ± 2 b	89 ± 1 ab
Cooking loss (g/100 g d.b.)	7.2 ± 0.3 b	9.7 ± 0.3 a	7.0 ± 0.5 b
Firmness (N)	597 ± 25 a	473 ± 37 b	584 ± 44 a
Starch digestibility			
TS (g/100 g w.b.)	25.3 ± 1.4 n.s.	24.7 ± 0.1 n.s.	24.2 ± 3.1 n.s.
RS (g/100 g w.b.)	2.3 ± 0.2 n.s.	2.0 ± 0.2 n.s.	2.1 ± 0.2 n.s.
RDS (g/100 g available starch)	59.3 ± 3.1 b	74.0 ± 4.5 a	72.8 ± 2.3 a
SDS (g/100 g available starch)	40.7 ± 3.1 a	26.0 ± 4.5 b	27.2 ± 2.3 b

Mean (*n* = 5 for cooking behaviour; *n* = 3 for starch digestibility) ± standard deviation followed by different letters in the same row are significantly different (Tukey’s HSD; *p* < 0.05). Separate ANOVA was carried out for different cooking times. n.s., not significant. d.b., dry basis. w.b., wet basis. C_R, pasta from raw red lentils by conventional extrusion; EC_R, pasta from raw red lentils by extrusion-cooking; C_HT, pasta from heat-treated red lentils by conventional extrusion. TS, total starch; RS, resistant starch; RDS, Rapidly digestible starch; SDS, Slowly digestible starch; Available starch, total starch minus resistant starch.

## Data Availability

The data used to support the findings of this study can be made available by the corresponding author upon request.

## Supplementary Materials

The following supporting information can be downloaded at: https://www.mdpi.com/article/10.3390/foods11244040/s1, Table S1: Chemical composition of experimental pasta samples from red lentils; Figure S1: SDS-PAGE of C_R, C_HT, and EC_R samples.
