# Supplementary material for: Pasta from Red Lentils (Lens culinaris): The Effect of Pasta-Making Process on Starch and Protein Features, and Cooking Behavior
Source: Foods. 2022 Dec 14;11(24):4040. doi: 10.3390/foods11244040 (PMC9778262; doi:10.3390/foods11244040)
Supplement: Supplementary file 1 [file foods-11-04040-s001.zip › foods-2086363-supplementary.pdf]

Supplementary material

Table S1. Chemical composition of experimental pasta samples from red lentils.

Samples	Moisture	Carbohydrates	Proteins	Lipids	Ash	Total fiber	Soluble fiber	Insoluble fiber
C_R	8.9	53.5	24.3	2.0	2.6	8.7	1.4	7.3
EC_R	8.8	53.9	24.8	2.3	2.5	7.7	1.1	6.6
C_HT	8.6	56.9	23.0	2.3	2.2	7.0	0.9	6.1

Data are expressed as g/100 g raw sample (wet basis).

C_R. pasta from raw red lentils by conventional extrusion; EC_R. pasta from raw red lentils by extrusion-cooking; C_HT. pasta from heat treated red lentils by conventional extrusion.

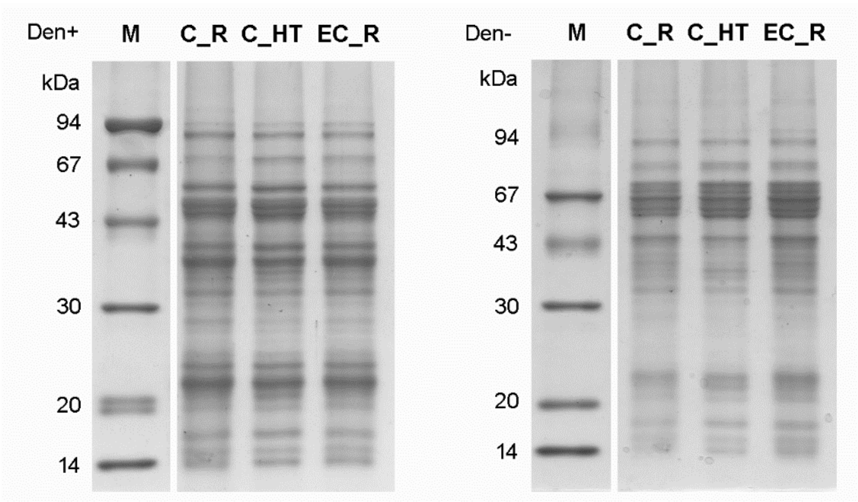

Supplementary Figure S1. SDS-PAGE of C_R, C_HT, and EC_R samples. Left, reducing conditions (Den+); right: non-reducing conditions (Den-). M, markers; C_R, pasta from raw red lentils by conventional extrusion; C_HT, pasta from heat treated red lentils by conventional extrusion; EC_R, pasta from raw red lentils by extrusion-cooking.
